# SHARED DECISION-MAKING WITH COST INFORMATION IN THE CONTEXT OF AGE-FRIENDLY CARE

**DOI:** 10.1093/geroni/igac059.1796

**Published:** 2022-12-20

**Authors:** Nicole Iny

**Affiliations:** FAIR Health, New York, New York, United States

## Abstract

The proposed poster session will explore shared decision making—the patient-clinician communication to decide on tests, treatment and care based on clinical evidence, balancing risks and outcomes with patient and caregiver preferences and values—and how evidence-based strategies, such as shared decision-making tools, or decision aids, can support older adults and their caregivers/care partners as they navigate the healthcare system. Notably, the session will share findings from FAIR Health’s grant-funded initiatives to advance shared decision making through decision aids, which combine clinical and cost information for specific clinical scenarios, and are freely available for consumers on fairhealthconsumer.org and for healthcare providers on fairhealthprovider.org. As such, the session will offer diverse perspectives and a unique spotlight on the financial and patient- and caregiver-centered aspects of healthcare decision making. Through FAIR Health’s needs assessment for a current project funded by The John A. Hartford Foundation, which has as its goal to advance shared decision making among older adults with serious illness and family caregivers, FAIR Health collected qualitative feedback from older patients, family caregivers and healthcare providers regarding perspectives on navigating the healthcare system. Through a national survey of older patients and family caregivers, FAIR Health found that: (1) though a significant proportion of older adults consider healthcare costs to be an important factor when making healthcare decisions, more than a third have difficulty getting such cost information; and (2) family caregivers/care partners expressed an appetite and need for healthcare information, resources and tools that facilitate better decisions about their care receiver’s care.

